# Time-resolved x-ray solution scattering from detergent solubilized visual rhodopsin

**DOI:** 10.1016/j.bpj.2026.01.014

**Published:** 2026-01-10

**Authors:** Daniel Sarabi, Lucija Ostojić, Xiaolin Xu, Thomas Gruhl, Niranjan Varma, Robert Bosman, Oskar Berntsson, Martin Nors Pedersen, Mathias Sander, Michael Wulff, Matteo Levantino, Gebhard F.X. Schertler, Michael F. Brown, Valerie Panneels, Richard Neutze

**Affiliations:** 1 Department of Chemistry and Molecular Biology, University of Gothenburg, Gothenburg, Sweden; 2 Department of Physics, University of Arizona, Tucson, Arizona; 3 Division of Biology and Chemistry, Laboratory for Biomolecular Research, Paul Scherrer Institute, Villigen PSI, Switzerland; 4 ESRF – The European Synchrotron, Grenoble, France; 5 Department of Chemistry and Biochemistry, University of Arizona, Tucson, Arizona

## Abstract

Time-resolved x-ray solution scattering (TR-XSS) studies provide experimental probes of transient conformational states in macromolecules. Difference x-ray scattering curves from integral membrane proteins are predicted to be influenced by the presence of the surrounding detergent micelle. Here, we present time-dependent x-ray solution scattering data from visual rhodopsin when solubilized in two different detergents: the nonionic surfactant *n*-dodecyl-β-D-maltoside and the zwitterionic detergent 3-[(3-cholamidopropyl) dimethylammonio]-1-propanesulfonate. Both detergents produce micelles that surround rhodopsin, yet they have different composition, density and critical micelle concentrations and yield different x-ray scattering properties. Our theoretical framework is able to fit the experimental TR-XSS data for photoactivated rhodopsin in both detergents, yielding experimental verification of how x-ray scattering contrast from the detergent molecules influences difference x-ray scattering measurements from integral membrane proteins. These results increase confidence when modeling conformational changes of integral membrane proteins from an ensemble of predicted structures.

## Significance

Biophysical methods that yield time-dependent structural data are essential for modeling transient conformations against experimental measurements. Time-resolved x-ray solution scattering (TR-XSS) provides a real-time probe of functional conformational changes of proteins and other macromolecules at ambient temperatures. Together with machine learning-based structural predictions, TR-XSS is expected to become increasingly useful for characterizing transient macromolecular conformations. TR-XSS data from visual rhodopsin validates the theoretical framework for modeling difference XSS data collected from integral membrane proteins. This framework will facilitate future structural modeling of transient conformations of membrane-bound macromolecules with diverse cellular functions.

## Introduction

Structural biology is experiencing a period of rapid change due to the success of machine learning algorithms for predicting protein structure (1,2). Despite very impressive recent progress, the protein data bank against which machine learning tools are trained is heavily biased toward resting state macromolecular structures. Because of the nature of these training sets, transient protein conformations cannot be predicted with the same level of confidence as resting conformations. Experimental probes that yield time-dependent structural data are therefore essential to constrain structural predictions for transient conformations against experimental measurements.

Time-resolved x-ray solution scattering (TR-XSS) of macromolecules provides a real-time probe of global conformational changes within macromolecules at physiological temperatures (3,4). One key advantage of TR-XSS relative to more information-rich methods, such as time-resolved x-ray diffraction (5,6), is that it is almost always straightforward to extract the number of protein conformations involving secondary structural changes, and their rise and decay times, directly from the experimental difference x-ray scattering data (3,4,7,8). Moreover, since TR-XSS studies are performed on proteins or other macromolecules in solution, there are no conformational constraints imposed by the crystal lattice, and hence secondary rearrangements can be characterized that may otherwise be inhibited within crystals. A particularly illustrative example of this concern are numerous low-temperature trapping and time-resolved x-ray diffraction studies of bacteriorhodopsin (9), for which an extended movement of helix F was suppressed in a large number of studies in the crystalline state. As such, a triple-mutant resting conformation (10,11) was required to guide structural modeling in the only time-resolved x-ray diffraction study to capture that motion (12). TR-XSS studies also utilized these triple-mutant structural models as guides to fit time-dependent x-ray scattering differences recorded from bacteriorhodopsin and thereby visualize the rise time and full extent of an outward motion of helix F (4,13).

In contrast with room temperature time-resolved x-ray diffraction methods (5) such as time-resolved Laue diffraction (14) and time-resolved serial x-ray crystallography (6,15), TR-XSS provides only one-dimensional information because x-ray scattering data are recorded from an ensemble of billions of molecules each with random orientation (16). As such, atomic coordinates cannot be optimized against three-dimensional experimental data. Additional structural information is therefore required, and structural fitting usually extracts candidate motions from crystallographic studies (4,17,18) or molecular dynamics simulations (18,19,20,21,22,23,24). For these reasons, rather than presenting unique structural solutions, structural analysis can confirm that candidate motions are consistent with the TR-XSS data. Structural results from TR-XSS have been reported from myoglobin (22,25,26,27), hemoglobin (3), bacteriorhodopsin (4), proteorhodopsin (28), visual rhodopsin (17), phytochromes (18,20,29), photosynthetic reaction centers (19), photoactive yellow protein (8,23), other photoreceptors (30), photoprotection proteins (31), during Ca^2+^ transport by the P-type ATPases (32,33), within a bacterial kinase (34), and during protein folding (35) and unfolding (36,37). A limitation of the field has been the requirement of a good model for the resting conformation before conformational changes can be modeled. With recent progress in the rate of structural determination by single-particle electron microscopy (38) and stunning improvements in the accuracy of protein structural prediction (1,2,39), this should no longer be a limiting factor when designing TR-XSS studies. Moreover, since protein structural prediction tools provide multiple conformational models, and single-particle electron microscopy studies often provide ensemble models (40), these data are also rich in terms of candidate motions against which TR-XSS data can be modeled. For these reasons, TR-XSS is expected to become a more important experimental tool in the future as increasingly reliable machine learning-based structural predictions emerge for resting macromolecular conformations, but with considerably less experimental data available concerning transient macromolecular conformations.

**Figure 1:**
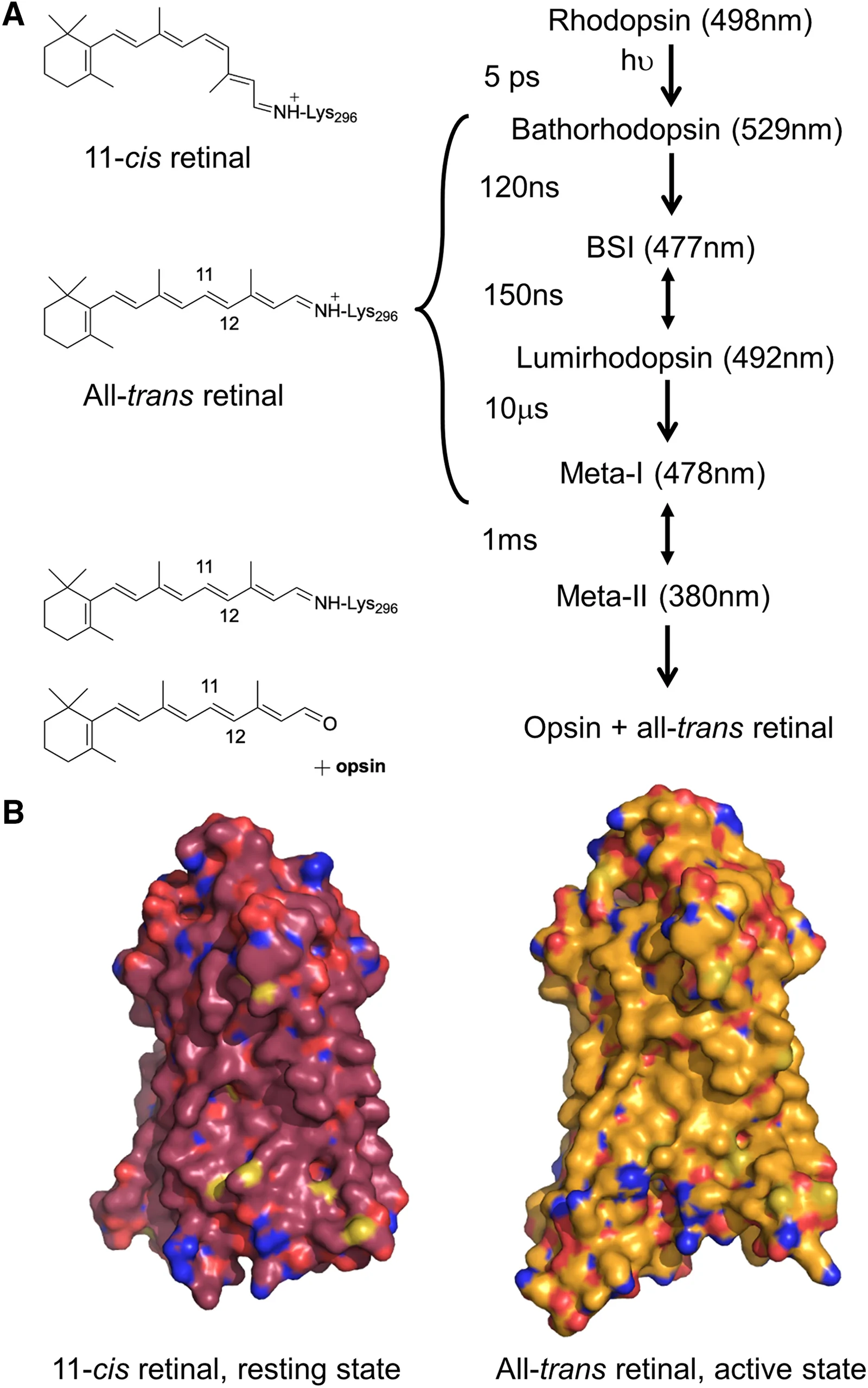
Light-initiated evolution of spectral and structural transitions in visual rhodopsin. (*A*) An absorbed photon (hν) causes 11-*cis* retinal (λ_max_ = 498 nm), which is covalently bound via a protonated Schiff base to Lys296 of TM7, to isomerize to an all-*trans* configuration. All-*trans* retinal is initially highly strained and relaxes through a sequence of spectral intermediates called bathorhodopsin (λ_max_ = 529 nm), the blue-shifted BSI intermediate (λ_max_ = 477 nm), and lumirhodopsin (λ_max_ = 492 nm) and Meta I (λ_max_ = 478 nm), all of which maintain the protonated Schiff base linkage to retinal. Structural transitions to the catalytically active Meta II state (λ_max_ = 380 nm) involves deprotonation of the Schiff base along with a large cytoplasmic conformational change. Meta II subsequently decays through an irreversible hydrolysis of the Schiff base linkage, releasing all-*trans* retinal and forming opsin. The retinal conformation is shown on the left and the approximate timescale of each spectral transition is indicated. (*B*) Surface representation of the resting state of rhodopsin with a compact structure, and the activated state which has undergone large helix rearrangements on the cytoplasmic side that initiate the binding of the G-protein transducin. These figures were drawn in PyMOL (43) using PDB: 7BZC (*dark*) and PDB: 4A4M (Meta II).

Integral membrane proteins are an important class of proteins that are critical for many cellular functions such as energy transduction and cellular signaling. One important class of integral membrane proteins are seven transmembrane α-helical G-protein-coupled receptors (GPCRs), of which approximately 800 GPCRs have been identified from human genome sequencing (41). GPCRs initiate signaling cascades in eukaryotes that allow cells to respond to stimuli such as light, neurotransmitters, hormones, and odorants. Activated GPCRs undergo large conformational rearrangements that lead to the binding of a heterotrimeric guanine nucleotide-binding protein (G-protein) with bound GDP. The exchange of GDP for GTP and subsequent dissociation of the α-subunit propagates and amplifies the signal (42). Rhodopsin is the light-receptor that is concentrated within disk membranes of photoreceptor rod cells and initiates vision in vertebrates under dim-light conditions. A single photon is absorbed by an 11-*cis* retinal chromophore that is covalently bound through a protonated Schiff base to Lys^296^ of TM7 (residues numbered according to bovine rhodopsin). This triggers retinal photoisomerization to an all-*trans* conformation, initiating a cascade of spectral (Fig. 1 *A*) and structural (Fig. 1 *B*) changes that lead to the formation of an activated state (metarhodopsin II) and to which the G-protein transducin binds (36,42). Activation of transducin then initiates a sequence of biochemical events that trigger a nerve impulse which is ultimately interpreted as vision in animals.

Structural characterization of integral membrane proteins usually requires that they are solubilized and purified using detergents or other amphiphilic molecules (44). Theoretical considerations have shown that the presence of a detergent micelle surrounding a protein will influence the difference x-ray scattering curves when comparing x-ray scattering data recorded from an activated conformation against data from the same sample in its resting conformation (13). In this work we explicitly test this prediction by recording TR-XSS data from light-activated samples of visual rhodopsin when solubilized in two different detergents, namely *n*-dodecyl-β-D-maltoside (DDM) and 3-[(3-cholamidopropyl) dimethylammonio]-1-propanesulfonate (CHAPS). We observe distinctly different x-ray scattering curves for the photoactivated state of rhodopsin in these two cases, because the detergent micelles of these two detergents yield very different x-ray scattering properties and have different solvent contrast. Nevertheless, the theoretical framework previously described (13) is able to produce good fits to these experimental data for both of these detergent molecules, thereby providing experimental verification of an important prediction concerning how x-ray scattering contrast from detergent-solubilized integral membrane proteins influences TR-XSS measurements. Overall, these findings increase confidence in the theoretical framework for modeling these data and will facilitate TR-XSS studies of other integral membrane proteins with diverse cellular functions.

## Materials and methods

### Sample preparation

Rod outer segments from disk membranes (RDMs) were isolated and purified from bovine retina (Lawson, Omaha, NE) under dim red light as previously described (45,46). Purified bovine RDMs typically had unregenerated *A*_280_/*A*_500_ absorption ratios <2.9 indicating high purity. The rhodopsin in RDMs was first solubilized in the cationic detergent dodecyltrimethylammonium bromide (DTAB) (Sigma-Aldrich, St. Louis, MO) and purified by hydroxyapatite (HA) column chromatography (46). After HA column purification the final *A*_280_/*A*_500_ ratio was <1.9 indicating highly pure rhodopsin. The detergent was then exchanged from DTAB to CHAPS by size-exclusion chromatography. The high-purity (*A*_280_/*A*_500_ < 1.9) fractions were pooled and concentrated using 30-kDa centrifugal filters, yielding a homogenous preparation. Rhodopsin samples in DDM were purified with the same level of quality (*A*_280_/*A*_500_ < 1.9) using a concanavalin A affinity column (47) after solubilizing the RDM with LDAO detergent and exchanging for DDM during elution from the affinity column. All samples were flash-frozen in liquid nitrogen and stored at −80°C until thawed just before use during the ESRF beamline. The final protein concentration was typically 20–30 mg mL^−1^ and a few hundred microliters of sample was required for both detergents in these studies.

### TR-XSS data acquisition and processing

TR-XSS data were collected at ID09 of the ESRF, a dedicated time-resolved x-ray scattering and x-ray diffraction instrument (48). Samples of visual rhodopsin were solubilized, purified, and concentrated to 20–30 mg/ml in either DDM or CHAPS. These samples were delivered across the polychromatic x-ray beam produced from an x-ray undulator (18 keV, pink beam, ΔE/E ≈ 4%, x-ray beam dimensions 60 *μ*m vertical × 100 *μ*m horizontal) through a 0.5-mm diameter glass capillary using a motorized syringe pump (neMESYS) with a flowrate of 2 or 3 *μ*L/s. This corresponds to a sample translation rate of 10 or 15 mm/s, respectively. TR-XSS data were collected using a repetition rate of 10 Hz at room temperature (∼20°C) under dim red-light conditions, corresponding to a translation of 1 or 1.5 mm between each x-ray exposure, respectively. Samples were photoactivated (laser on) perpendicular to the x-ray beam (spot size 290 × 700 *μ*m full-width half maximum) using a 5-ns duration laser pulse of 532 nm and a total power of 200 *μ*J/pulse. This corresponds to a power density *F* = 44 mJ/cm^2^ averaged throughout the focal spot and has a maximum value of 61 mJ/cm^2^. These values are comparable with those used in time-resolved serial x-ray crystallography studies of light-sensitive molecules (5). However, the ratio σ_530nm_ **×** F/h‧ν ≈ 40, where σ_530nm_ = 90,000 M^−1^·cm^−1^ is the absorption cross section of rhodopsin at 530 nm, *h* is Planck’s constant, and ν is the frequency of the incoming light. Accounting for the optical density of the sample (∼0.8–1.2 over the 0.5-mm diameter capillary) suggests the order of eight or less photons are absorbed per chromophore on average, which is in agreement with the value of seven absorbed photons per chromophore estimated from the observed sample heating. As such, retinal was photoexcited multiple times during the 5-ns laser pulse. The trade-off between using single-photon excitation but potentially achieving only a small fraction of molecules in the photoactivated state, and using excessive pump laser fluence but risking multiphoton effects, has been heavily debated (49,50). Current structural evidence suggests that multiphoton effects may be detectable only in the ultrafast domain (51).

X-ray pulse trains of 1 or 5 *μ*s in duration were isolated from a continuous filling of the storage ring using an x-ray chopper, with the arrival of the visible laser pulse relative to the x-ray pulse train controlled electronically. The x-ray scattering images were recorded on a Rayonix MX170-HS detector (1920 × 1920 pixels, pixels binned 2 × 2 to a pixel-size of 88.5 *μ*m) located 350 mm from the sample position. Reference data (laser off) were recorded in exactly the same manner, but with the laser beam blocked by a shutter such that no laser pulse reached the sample position and therefore could not photoactivate the sample. X-ray scattering data were integrated in concentric rings before normalization about the isosbestic points at *q* = 2.2–2.3 Å^−1^. Absolute scattering curves were first rejected if they deviated more than 10% from the median value in the *q*-range 2.0–2.5 Å^−1^, and a second outlier rejection criteria was applied if the difference x-ray scattering curves (ON minus OFF) deviated by more than three standard deviations from the mean of the set. Typically, 5–10% of all the data were thereby rejected using these criteria. Each time point in the final data sets was averaged over approximately 75 difference scattering curves, corresponding to different 7500 x-ray exposures since each x-ray image was read out after 100 repeats. Since visual rhodopsin does not return to its resting state, samples were discarded after each exposure to green light. The influence of laser-induced heating was measured and removed (Fig. 3 *B*) using data recorded from visual rhodopsin solubilized in CHAPS, and the pump laser beam was aligned both below and above the x-ray beam in two separate measurements. From this combination, a pure heating curve was extracted and heat-free light-induced difference scattering data (Fig. 4, *A* and *B*) were computed by scaling and removing the thermal signal from each time point.

### Linear decomposition of the TR-XSS data

Heat-free data (Fig. 4, *A* and *B*) were analyzed using linear decomposition by applying a two-component fitting assuming an exponential decay of the first basis spectrum component (BS1), with a corresponding exponential rise of the second component (BS2). This procedure indicated a decay time of 350 *μ*s for the first component of BS1_DDM_ and a decay time of 8.5 ms for BS1_CHAPS_. Since photoactivated rhodopsin does not return to the resting state and the metarhodopsin II state is believed to be stable for up to minutes, the time of decay for the second component reflects the time for the photo-exposed sample to be swept away from its position of overlap with the x-ray beam.

### Structural refinement of the XSS signal

**Figure 2:**
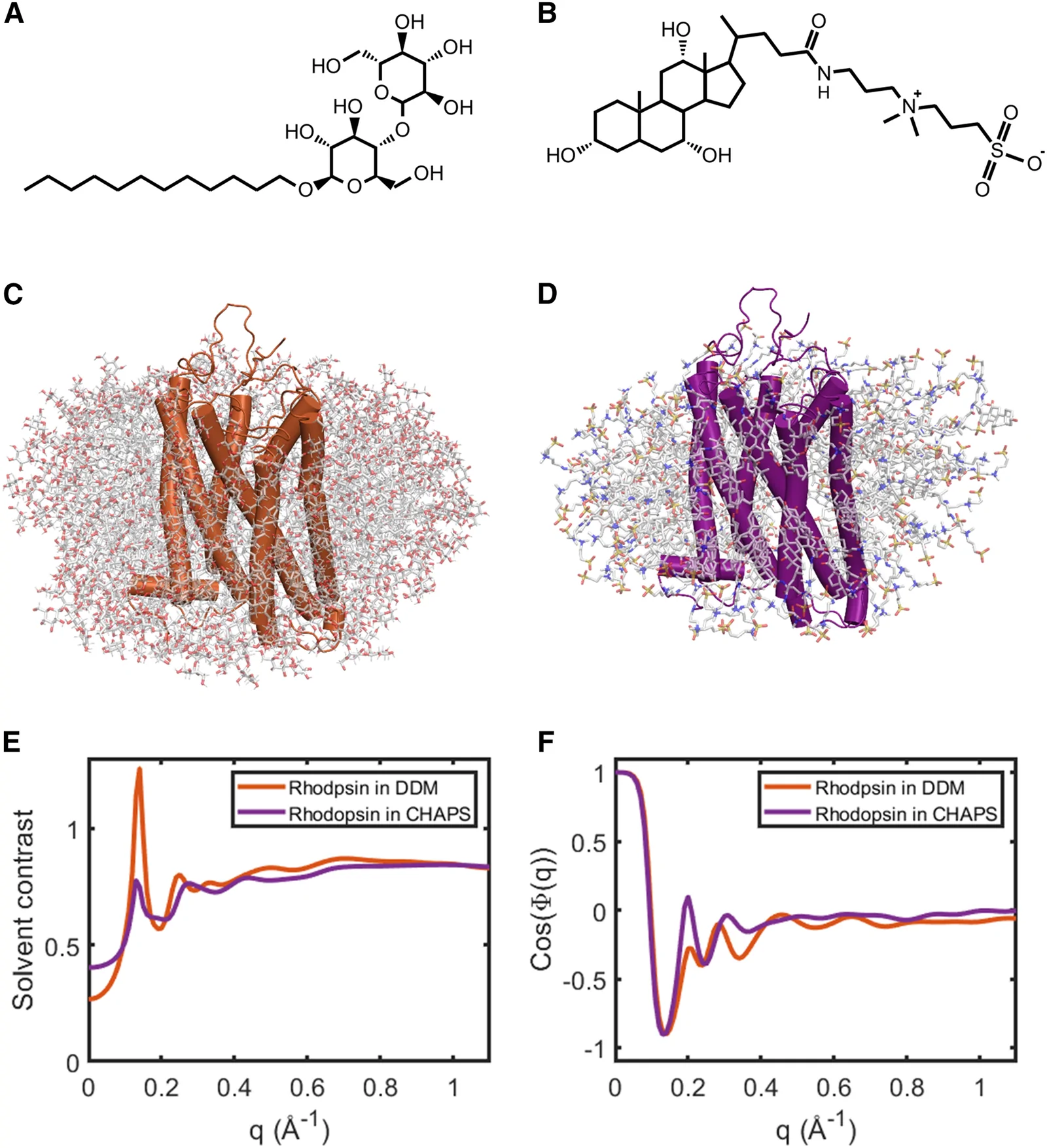
Chemical formula, arrangement, and x-ray scattering properties of detergent molecules used to solubilize visual rhodopsin. (*A*) *n*-Dodecyl-β-D-maltoside (DDM) with molecular weight 511 Da. (*B*) 3-[(3-Cholamidopropyl) dimethylammonio]-1-propanesulfonate (CHAPS) with molecular weight 615 Da. (*C*) Structure of visual rhodopsin in a micelle containing 215 molecules of DDM used for molecular dynamics simulations. (*D*) Structure of visual rhodopsin in a micelle consisting of 130 molecules of CHAPS used for molecular dynamics simulations. (*E*) Solvent contrast correction, defined as the ratio of the total x-ray scattering divided by the x-ray scattering from the protein-micelle system in vacuum ((*S*_*tot*_(*q*)/*S*_*pm*_(*q*)) (defined in Eqs. 9 and 11 of (13)). (*F*) Cosine of the phase factor, cos(*Փ*(*q*)), describing the effective phase relationship between x-ray scattering from the protein and surrounding detergent micelle (defined in Eq. 6 of (13)). Differences between the x-ray scattering properties of these detergent micelle systems lead to the measured difference x-ray scattering data recorded from rhodopsin in DDM and CHAPS micelles.

Visual rhodopsin was built into a detergent micelle using the CHARMM-GUI Micelle Builder (52,53) (version Charmm36-jul2020). These micelle complexes contained 215 DDM molecules (Fig. 2 *C*) and 130 CHAPS molecules (Fig. 2 *D*). Detergent ring and protein surface penetration was checked by CHARMM as part of the system building options. The system was built within a solvent box consisting of 20 Å in all dimensions, using the three-site CHARMM modified TIP3 water model and a concentration of 0.15 M of NaCl. GROMACS input files were created by CHARMM (54). GROMACS 2019.1 (55,56) was used to run simulations of visual rhodopsin within the micelle using the CHARMM36 force field (57). Energy minimization was carried out first using a LINCS constraints algorithm with constraints on hydrogen bonds. The second stage was equilibration of the system in six steps with energy and dihedral restraints on the detergent molecules.

An ensemble of protein:detergent micelle conformations was achieved through molecular dynamics simulations using GROMACS, with the protein driven toward a target structure given by Eq. 3: ${C\alpha }_{SRII}\to {C\alpha }_{SRII}+{{\gamma \times \Delta C\alpha }_{TM5,6}+\delta \times \Delta C\alpha }_{TM7}$, where the α-helix perturbation Δ*Cα*_*TM*5,6_ was calculated from the Cα coordinates of residues Leu^216^ (TM5) to Cys^264^ (TM6) from PDB: 3PXO (58) minus those of PDB: 1GZM (59), and Δ*Cα*_*TM*7_ was calculated from the Cα coordinates of residues Ile^286^ (TM7) to Asn^302^ (TM7) of PDB: 3CAP (60) minus those of PDB: 1GZM (59). Nine values of *γ* (from 0 to 8/3) and four values of *δ* (from 0 to 1) were used to scale these candidate motions, giving 36 simulated candidate trajectories in total, each with retinal isomerized to an all-*trans* configuration. The γ domain had to be extended by adding simulations until a minimum *R*-factor was reached (Fig. 6), whereas there was almost no sensitivity in the *R*-factor to changes in δ and therefore the smaller domain was sufficient. For each value of *γ* and *δ*, and a control trajectory in which the retinal remains in its 11-*cis* configuration, a simulated trajectory was performed with the protein embedded in a detergent micelle and restrained about the conformation specified by *γ* and *δ*, and 501 evenly spaced coordinates for the protein and detergent micelle were written to disk from a simulation 5 ns in duration. X-ray scattering curves were then calculated from these coordinates for the protein alone, the micelle alone, and the protein plus detergent micelle, using CRYSOL (61). A complementary set of PDB files was created by swapping the coordinates of the detergent micelle between the target (all *trans*, *γ* and *δ* varying) and the control trajectories (11-*cis*, resting conformation), and this manipulation was used to cancel the effects of structural fluctuations in the detergent micelle in the difference x-ray scattering calculations (13). SVD was used to extract an x-ray scattering spectrum characterizing the influence of structural fluctuations within each detergent micelle on the difference x-ray scattering, Δ*S*_*mc*_(*q*), and this was incorporated into structural fitting as a low-resolution correction term (13). The influence of the solvent excluded volume of the protein-micelle system was also calculated as described using the same molecular dynamics trajectories.

From the set of 501 trajectories for any given values of *γ* and *δ*, 501 difference x-ray scattering curves were calculated and a Pearson correlation score was calculated against the experimental difference curves over the domain 0.3 Å^−1^ ≤ *q* ≤ 1.0 Å^−1^. From this set, the 50 pairs with the highest correlation were kept for further analysis. A weighted *R*-factor (residual difference between the theoretical prediction and experimental data expressed as a percentage) was calculated over the domain 0.13 Å^−1^≤*q* ≤ 1.0 Å^−1^ for every value of *δ* and *γ*:

(1) $$R=\frac{\sum \sqrt{w^{2}\left(q\right)\cdot {\left({A_{1}‧\Delta S}_{theory}\left(q\right)-{\Delta S}_{expt}\left(q\right)\right)}^{2}}}{\sum \sqrt{w^{2}\left(q\right)\cdot {\left({\Delta S}_{expt}\left(q\right)\right)}^{2}}}\text{,}$$

where Δ*S*_*expt*_(*q*) is the experimental basis difference spectrum (Fig. 1, *E* and *F*) and *w*(*q*) is a weighting function introduced to emphasize difference x-ray scattering around *q* ∼0.4 Å^−1^. If *w*(*q*) is chosen to equal 1 for all values of *q* then a quantity similar to the usual *R*-factor associated with x-ray crystallography is recovered. In this case, however, without the weighting factor the *R*-factor recovered for BS2_CHAPS_ after structural modeling is unexpectedly low (13.5 vs. 24.6% for BS2_CHAPS_, compared with 23.4 vs. 22.7% for BS2_DDM_) due to the very good agreement between experiment and theory at low *q*. An overall scaling factor *A*_1_ was optimized to give the best fit for data recorded using DDM and data recorded using CHAPS. For every value of *γ* and *δ*, the theoretical prediction Δ*S*_*theory*_(*q*) of Eq. 1 is calculated using the following expression:

(2) $${\Delta S}_{theory}\left(q\right)=\frac{S_{tot}\left(q\right)}{S_{pm}\left(q\right)}‧\left[\Delta S_{p}\left(q\right)+\frac{1-\mathrm{erf}\left(q_{pm},{\Delta q}_{pm}\right)}{2}\times (\Delta S_{pm}\left(q\right)-\Delta S_{p}\left(q\right)+A_{2}‧\;\Delta S_{mc}\left(q\right))\right]\text{,}$$

where the ratio $S_{tot}\left(q\right)/S_{pm}\left(q\right)$ corrects for the influence of the solvent excluded volume by comparing GROMACS simulations of the protein-micelle system against simulations of water alone, as was described previously (13). The error function, erf(*q*_*pm*_,Δ*q*_*pm*_), is used to constrain to low-resolution the contribution to the difference x-ray scattering of both the protein-micelle cross term and a detergent micelle alone, and its midpoint (*q*_*pm*_)and width (Δ*q*_*pm*_)were optimized independently. The second scaling coefficient, *A*_2_, allows for low-angle fluctuations in the micelle and is optimized for each data set. The protein-in-vacuum term, Δ*S*_*p*_(*q*), is predicted from the atomistic molecular dynamics simulations of the protein in a micelle with energy constraints to drive the protein toward the target structure, and fluctuations about this structure are sufficient to predict the damping of oscillating features in Δ*S*_*expt*_(*q*) at higher *q* values without the need to introduce a *B*-factor (4). Best-fit solutions resulting from this analysis are shown as contour plots in Fig. 6.

Coordinate uncertainty bars were estimated from this fitting protocol utilizing a Boltzmann weighting factor = $\text{exp}\left(\frac{-R-R_{\min}}{k\times R_{\min}}\right)$, where *R* is the *R*-factor calculated from Eq. 1 from each set of trajectories with a specified value of *γ* and *δ*, and the scaling factor *k* is chosen to be approximately 20% of *R*_*min*_, where *R*_*min*_ is the minimum weighted *R*-factor recovered for each basis spectrum: BS1_DDM_, BS2_DDM_, BS1_CHAPS_, and BS2_CHAPS_. From the set of 50 pairs of structures for each combination of *γ* and *δ*, a subset of these 50 structural pairs were kept with that specific fraction determined by the Boltzmann weighting factor $\text{exp}\left(\frac{-R-R_{\min}}{k\times R_{\min}}\right)$. The rmsd of Cα coordinates of the perturbed conformations from the control conformations were calculated, the mean value of this set of rmsd perturbations of Cα coordinates were calculated, and the standard deviation this set of Cα perturbations were calculated. The results of this analysis are plotted as the mean rmsd of Cα atoms with corresponding error bars in Fig. 7, *A*–*C*. The best-fit models are represented as protein conformational changes in Fig. 7, *D*–*F*, which was drawn in PyMOL (version 2.5.8) (43).

## Results

### TR-XSS, data collection, and spectral decomposition

Samples of visual rhodopsin were prepared in the dark as described (17,36). Detergent solubilization was performed using two detergent molecules, DDM and CHAPS. The DDM molecule (Fig. 2 *A*) is a nonionic surfactant that has proven advantageous when extracting integral membrane proteins from biological membranes and during protein purification. Notably DDM has a 12-carbon chain which provides a hydrophobic region surrounding the hydrophobic belt of the integral membrane protein. It contains a polar maltoside headgroup that is exposed to the surrounding solvent and thereby provides solubility. On the other hand, CHAPS (Fig. 2 *B*) has both ammonium and sulfonate groups, making this detergent zwitterionic, which means it has an equal number of positive and negative charges. CHAPS is structurally similar to some bile acids and is effective for membrane protein solubilization. Both DDM and CHAPS form relatively large detergent micelles (Fig. 2, *C* and *D*) but their different chemical composition, critical micellar concentration (0.17 and 8 mM, respectively, (62)), and average electron density lead to different x-ray scattering properties. These include different solvent contrast when used to solubilize integral membrane proteins, which was calculated (Fig. 2 *E*) by comparing molecular dynamics simulations of the protein and detergent in solution against a parallel simulation containing only water molecules (13). Moreover, the manner in which the x-ray scattering from the detergent and the protein interfere is different for each detergent. This we have characterized according to the parameter *cos*(*Φ*(*q*)) (Eq. 6 of (13)) and this is positive when this interference is constructive (and therefore equals unity in the forward direction, where *q* = 0) negative when their mutual interference is destructive, and approaches zero at higher x-ray scattering angle due to interreference effects (Fig. 2 *F*). These different properties predict that TR-XSS measurements will depend not only upon the conformational changes in rhodopsin but also on the choice of detergent (13).

**Figure 3:**
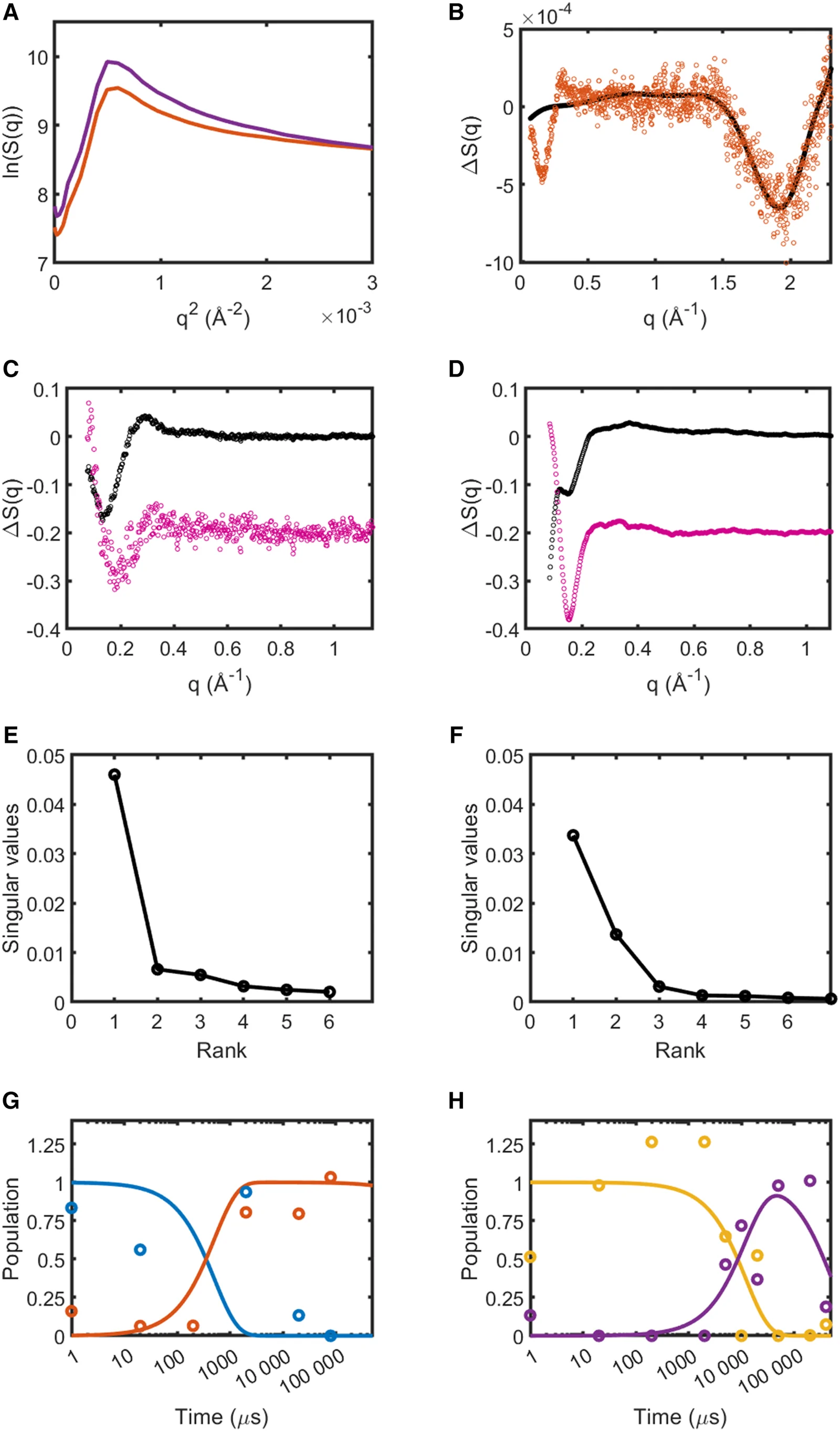
Analysis of TR-XSS data. (*A*) Guinier plot (ln(*S*(*q*)versus *q*^2^) illustrating how the x-ray scattering at low angles tends upward, indicating potential aggregation of the sample at the high concentrations used in this study. In both cases the x-ray scattering falls off below q^2^ ≤ 0.8 × 10^−3^ Å^−2^ due to the presence of the x-ray backstop. (*B*) Removal of the experimental heating from the TR-XSS data before further analysis. A difference x-ray scattering curve associated with sample heating alone (*black*, modeled as a high-order polynomial) is scaled to overlap with the experimental TR-XSS data (*Δt* = 200 *μ*s is illustrated for DDM) and subtracted, to recover the heat-free data shown in Fig. 4*A* and *B*. (*C*) The principal (*black*) and second (*magenta*) basis spectra, U_i_, following singular value decomposition (SVD) of the heat-free data for visual rhodopsin solubilized in DDM shown in Fig. 4*A*. (*D*) The principal (*black*) and second (*magenta*) basis spectra, U_i_, following SVD of the heat-free data for visual rhodopsin solubilized in CHAPS shown in Fig. 4*B*. (*E*) The singular values of the first six components of SVD analysis of the heat-free data for visual rhodopsin solubilized in DDM. (*F*) The singular values of the first six components of SVD analysis of the heat-free data for visual rhodopsin solubilized in CHAPS. (*G*) Amplitudes associated with BS1_DDM_ (*blue circles*) and BS2_DDM_ (*red circles*) derived from a linear reconstruction of the TR-XSS data (Fig. 4*A*). These values are superimposed upon the time-dependent evolution of the population of each component used to decompose these data (Fig. 4*E*). (*H*) Amplitudes associated with BS1_CHAPS_ (*blue circles*) and BS2_CHAPS_ (*red circles*) derived from a linear reconstruction of the TR-XSS data (Fig. 4*A*). These values are superimposed upon the time-dependent evolution of the population of each component used to decompose these data (Fig. 4*F*).

In the present studies, TR-XSS data were collected from concentrated solutions (∼20–30 mg/ml) of bovine rhodopsin solubilized and purified using both DDM and CHAPS. At these high concentrations, x-ray scattering at low-angle deviates from linearity in a Guinier plot (Fig. 3 *A*), indicating some sample aggregation. This illustrates a trade-off required in TR-XSS studies, since if the protein concentration is too low the difference x-ray scattering signal becomes difficult to extract with an acceptable signal/noise ratio, whereas at higher protein concentrations problems associated with sample aggregation may arise. Data were collected at beamline ID09 of the European Synchrotron Radiation Facility (ESRF). This station is a dedicated time-resolved x-ray diffraction and x-ray scattering instrument that allows individual or trains of polychromatic x-ray pulses to be isolated using an x-ray chopper, and their arrival to be synchronized relative to a pump laser pulse usually operating in the infrared, visible, or ultraviolet wavelength domains (48). Because the light-initiated reactions of visual rhodopsin are irreversible (Fig. 1), samples could not be cycled and the amount of data collected was limited by protein supply.

When recording TR-XSS data from visual rhodopsin solubilized in DDM, we used the time delays Δ*t* = 1 *μ*s, 20 *μ*s, 200 *μ*s, 2 ms, 20 ms, and 80 ms between the arrival of a green laser pulse (λ = 532 nm) and the x-ray pulse train, which was 5 *μ*s in duration, except for the time delay of Δ*t* = 1 *μ*s for which the pulse train was also shortened to be 1 *μ*s in duration. When recording TR-XSS data from rhodopsin solubilized in CHAPS we used the time delays Δ*t* = 1 *μ*s, 20 *μ*s, 200 *μ*s, 2 ms, 5 ms, 10 ms, 20 ms, 50 ms, 200 ms, and 400 ms. For the longer time delays, it was necessary to offset the position of the visible (green) laser pump to compensate for the distance traveled by the sample after the laser flash and before the arrival of the x-ray pulse train. As with previous TR-XSS studies of light-sensitive proteins (3,4), the absorption of light causes a heating signal that is visible at higher x-ray scattering angle (Fig. 3 *B*) as the buffer (consisting primarily of water) expands (adopts a lower density), which causes characteristic oscillations either side of the water x-ray scattering peak from *q* = 1.5–2.2 Å^−1^, where $q=\frac{4\pi \;\sin\left(\theta \right)}{\lambda }=\frac{4\pi }{2d}$ and $\frac{1}{d}$ is the resolution usually quoted in x-ray crystallography, and *λ* is the wavelength of the x rays in Å. Accordingly, heating measurements were performed with the laser beam offset relative to the x-ray sample so that different ratios of sample heating to protein signal were recorded, and the pure heating term was then separated from the protein’s structural signal, modeled as a high-order polynomial, and subtracted from the time-dependent difference x-ray scattering data (Fig. 3 *B*). Calibrating these heating corrections against XSS data from a membrane protein as its temperature was raised (19) suggests that the laser-driven temperature jump was approximately 0.2°C, which corresponds to approximately seven incoming photons absorbed per rhodopsin chromophore.

**Figure 4:**
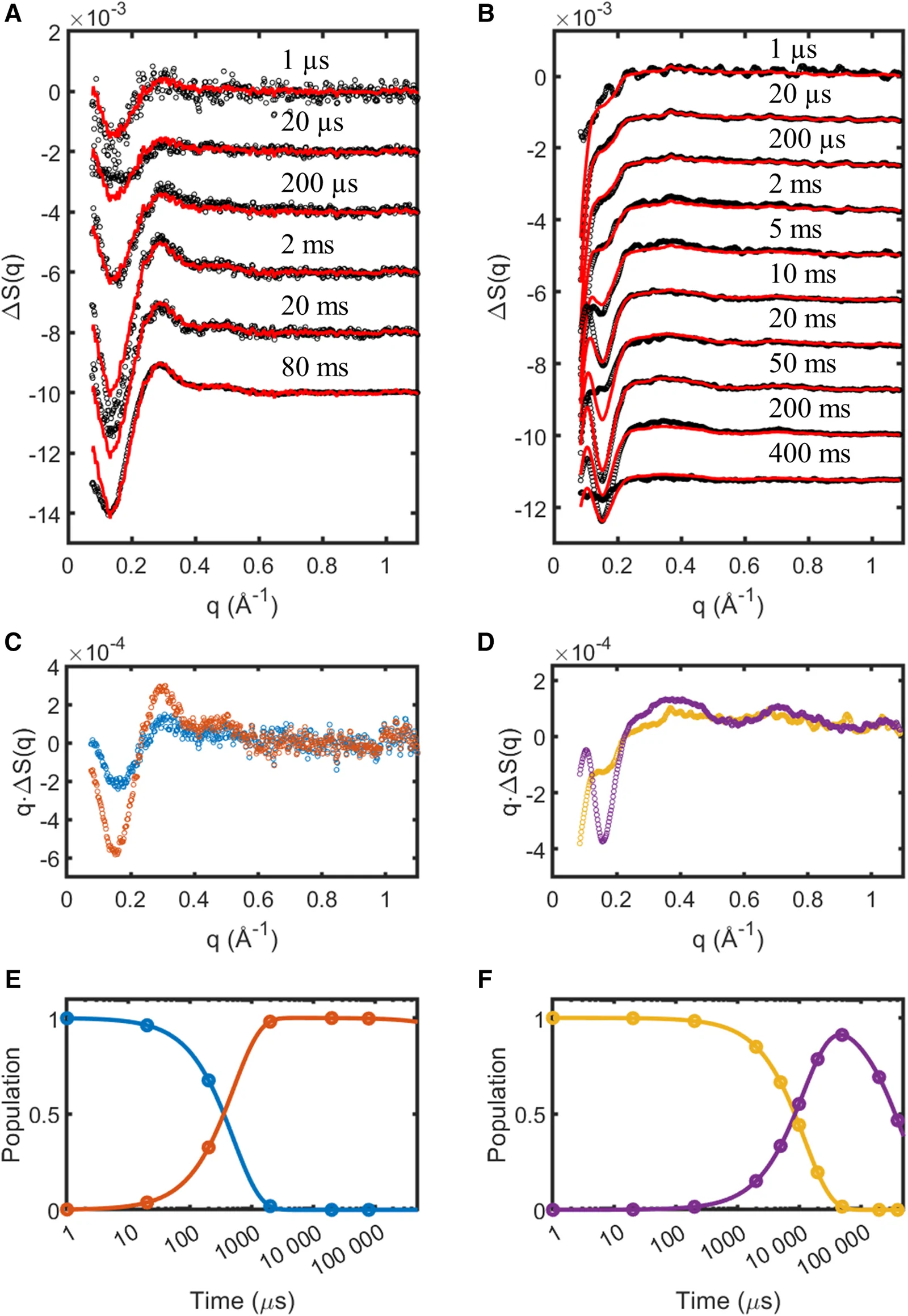
Time-resolved x-ray solution scattering difference data following the photoactivation of visual rhodopsin. (*A*) Time-dependent difference x-ray scatting curves recorded from samples of rhodopsin solubilized in DDM. (*B*) Time-dependent difference x-ray scatting curves recorded from samples of rhodopsin solubilized in CHAPS. For both panels the black circles show the experimental data and the red lines show their reconstruction from the basis spectra (*C* and *D*) recovered by linear decomposition of the populations of these basis spectra as a function of time (*E* and *F*). (*C*) Basis spectra showing both the early and late component (BS1_DDM_ in *blue*; BS2 _DDM_ in *red*) extracted by linear decomposition of the TR-XSS data obtained for rhodopsin in DDM detergent micelles. (*D*) Basis spectra showing both the early and late component (BS1_CHAPS_ in *mustard*; BS2_CHAPS_ in *purple*) extracted by linear decomposition of the TR-XSS data obtained for rhodopsin in CHAPS detergent micelles. (*E*) Time-evolution of the populations of the basis spectra showing the early component (BS1_DDM_, *blue*) and later component (BS2_DDM_, *red*) extracted by linear decomposition of TR-XSS data recorded from rhodopsin solubilized in DDM. (*F*) Time-evolution of the populations of the basis spectra showing the early component (BS1_CHAPS_, *mustard*) and later component (BS2_CHAPS_, *purple*) extracted by linear decomposition of TR-XSS data recorded from rhodopsin solubilized in CHAPS detergent micelles.

TR-XSS difference data (Δ*S*(*q*,Δ*t*)) after the influence of sample heating is removed are shown in Fig. 4 for rhodopsin solubilized in DDM (Fig. 4 *A*) and CHAPS (Fig. 4 *B*), respectively. For both sets of collected TR-XSS data, the difference curves reveal time-dependent changes in the x-ray scattering amplitudes over the domain 0.10 Å^−1^ < *q* < 0.80 Å^−1^. These features are characteristic of rearrangements of α-helices occurring in retinal proteins (4,13,17,28,63). Singular value decomposition (SVD) analysis of these data suggests that there are two components associated with the time evolution of visual rhodopsin in CHAPS (Fig. 3, *D* and *F*) and one predominant component associated with the time evolution of visual rhodopsin in DDM (Fig. 3, *C* and *E*). Using a procedure previously developed for studies of bacteriorhodopsin and proteorhodopsin (4), we decomposed the time-dependent XSS signal into an evolution of two components with time-dependent amplitudes. This model assumes an initial population of state 1 that decays exponentially into state 2, and state 2 has a complementary rise in population before decaying on a slower timescale (see Eqs. 2 and 4 of the supplemental information of (4)). This procedure yields two rate constants for each detergent and two basis spectra, where BS1_DDM_ and BS2_DDM_ (Fig. 4 *C*) are the first and second basis spectra recovered from rhodopsin in DDM, and BS1_CHAPS_ and BS2_CHAPS_ (Fig. 4 *D*) are the basis spectra recovered from rhodopsin in CHAPS. Each of these basis spectra are recovered as an optimized linear combination of the first two SVD components (Fig. 3, *C* and *D*) given constraints on the populations of each state defined by the time-dependent populations predicted by the model (Fig. 4 *E* for DDM; Fig. 4 *F* for CHAPS).

From this linear decomposition of the TR-XSS data it is observed that the amplitude of oscillations in the difference XSS data for *q* ≥ 0.2 Å^−1^ are larger in BS2 than in BS1, and sharper features emerge in the low-q domain *q* ≤ 0.2 Å^−1^, where this region is known to be heavily influenced by the presence of the detergent micelle (13,64,65). It is also apparent from this decomposition that the time-evolution of the structural signal differs between the two detergent molecules. More specifically, the transition from the early to late basis spectrum occurs with a midpoint at Δ*t* ≈ 350 *μ*s when rhodopsin is solubilized in DDM (Fig. 4 *E*), whereas this transition occurs for Δ*t* ≈ 8.5 ms in CHAPS (Fig. 4 *F*). Previous TR-XSS studies of the evolution of structural changes of visual rhodopsin in its native membrane environment (17) suggested a transition time to the latter conformation of Δ*t* ≈ 13 ± 5 ms, which is consistent with that recovered from rhodopsin in CHAPS detergent micelles. Moreover, a rise time of approximately 10 ms for CHAPS micelles correlates with the rise time of the metarhodopsin II photointermediate in disk membranes at room temperature, which has been measured using both absorption (66,67) and fluorescence (68,69) spectroscopy. As such, our data thus suggest that the conformational dynamics of the transition to metarhodopsin II of visual rhodopsin is accelerated by more than an order of magnitude when solubilized in DDM, which is consistent with optical spectroscopy studies that also suggest that metarhodopsin II forms in a few hundred microseconds in DDM (70).

As with studies of rhodopsin in its native membrane (17), our TR-XSS data from rhodopsin in DDM and CHAPS do not imply a decay of the metarhodopsin II state over the time-window of our data. Although there is an apparent decrease in the population of BS2 for the longest time delays of 200 and 400 ms recorded when using CHAPS to solubilize rhodopsin (Fig. 4 *F*), this observation reflects the effects of the light-illuminated region flowing outside of the area probed by the x-ray beam rather than indicating a structural evolution from the metarhodopsin II state, which is known to be stable for up to minutes. More specifically, photoactivated bovine rhodopsin does not return to its starting conformation because the covalent bond through a Schiff base linking retinal to Lys^296^ eventually becomes hydrolyzed (Fig. 1 *A*) and releases retinal from the rhodopsin binding pocket (42). The smaller maximum time-delay of 80 ms used for measurements using DDM to solubilize visual rhodopsin, meant that the effect of the illuminated sample moving outside of the x-ray probe was not as obvious in those data. Finally, limitations in the quality of the above linear decomposition can be seen by performing a linear fit of BS1 and BS2 for each detergent to the difference XSS data associated with each time delay (Fig. 3, *G* and *H*). This decomposition suggests a time-dependent growth in the population of BS1_CHAPS_ is present in these data that is not incorporated into the assumptions of the linear decomposition. However, a shift to a more complex kinetic model that could describe this effect would require that more time delays were sampled and that the signal/noise ratios in these data were improved. This, ultimately, is limited by the amount of sample that can be produced since it was not possible to repeatedly cycle visual rhodopsin due to the light-driven reaction being irreversible (Fig. 1 *A*).

### Structural fitting of conformational changes in visual rhodopsin

**Figure 5:**
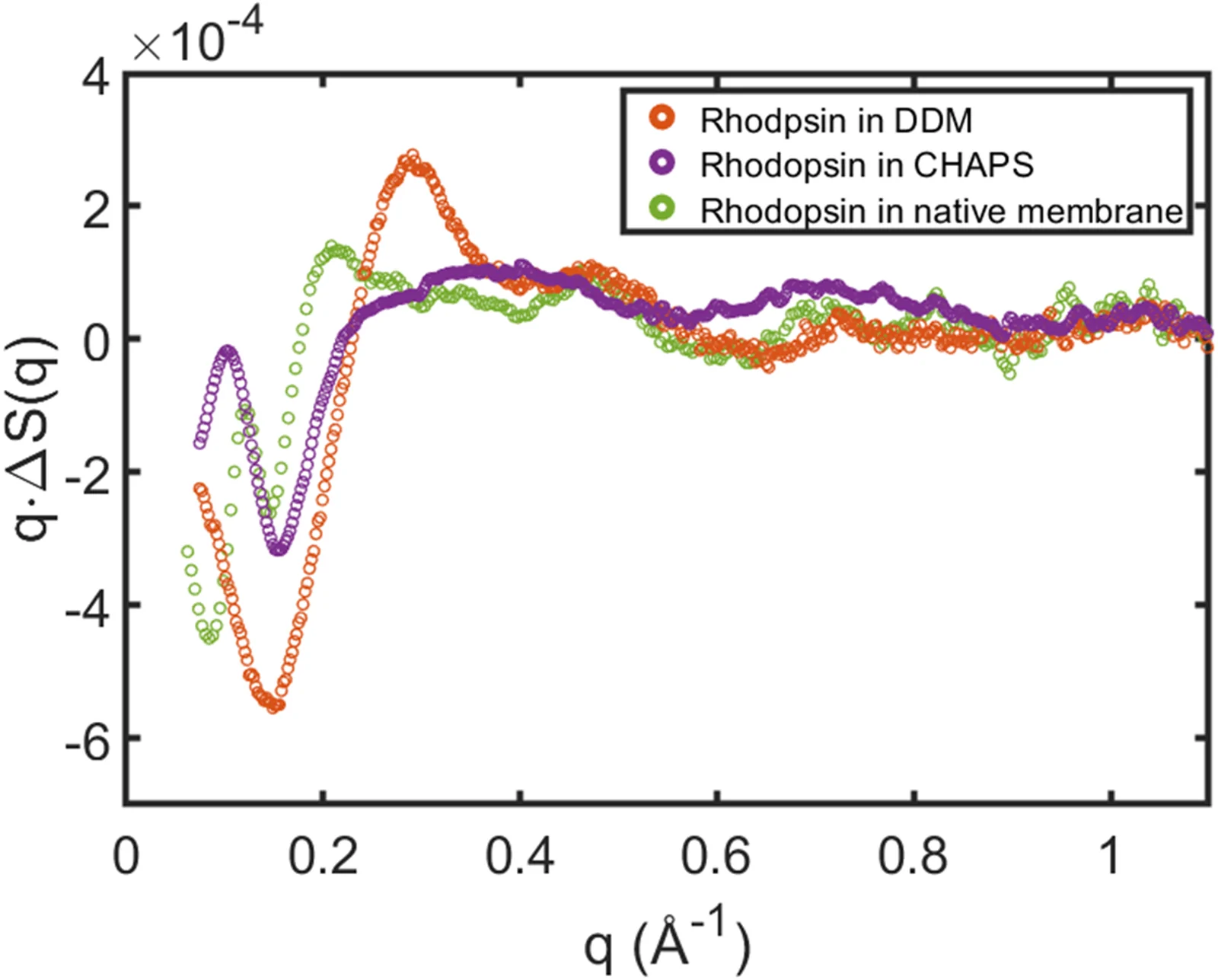
Comparison of the difference x-ray scattering curves recorded from rhodopsin extracted by linear decomposition of TR-XSS data. Overlay of BS2_DDM_ (*red*), BS2_CHAPS_ (*purple*), and the basis spectrum extracted from TR-XSS studies of visual rhodopsin in native membranes (*green*) (17). This comparison illustrates how the protein’s surroundings influence the difference x-ray scattering.

Fig. 5 overlays BS2_DDM_ recorded from rhodopsin in DDM, BS2_CHAPS_ recorded from rhodopsin in CHAPS, and the basis spectrum measured from photoactivated samples of rhodopsin in native membranes (17). These basis spectra are very distinct, with a prominent positive peak visible in the DDM data near *q* ≈ 0.30 Å^−1^ which is absent in the data from CHAPS solubilized rhodopsin, whereas a similar feature is shifted to lower *q* values in native disk membranes. There are also shifts in the position of peaks and valleys of these difference XSS data along the *q* axis. These data therefore confirm theoretical predictions that difference x-ray scattering data from integral membrane proteins will be strongly influenced by the properties of the medium that surround the protein (13).

As with all previous TR-XSS analyses of rhodopsins (4,13,17,28,63), we utilized additional structural information to model conformational changes against these difference XSS data. Visual rhodopsin was the first GPCR to have its structure solved at high resolution (71) and numerous experiments have revealed substantial movements in the cytoplasmic portions of TM5 and TM6 occurring following photoactivation, including electron paramagnetic resonance spectroscopy (72,73), fluorescence spectroscopy (74,75) and Fourier transform infrared spectroscopy (76,77,78). Although rhodopsin is believed to form dimers in the native membrane (79,80), we modeled conformational changes in DDM and CHAPS detergent micelles using monomeric rhodopsin since the process of detergent solubilization and purification may reasonably be expected to break the dimer. Previous modeling of light-induced conformational changes in visual rhodopsin against TR-XSS data (17) systematically tested candidate motions extracted from low-temperature intermediate trapping x-ray crystallography studies of rhodopsin, or structures of specific mutants that induce conformational changes believed to mimic rhodopsin intermediate states (58,60,81,82,83,84,85,86,87,88,89,90). That analysis built from crystallographic work for which the largest structural rearrangements were visible when the resting conformation of rhodopsin was compared against two metarhodopsin II structures (58,88), the apoprotein opsin structure (60), or a constitutively active structure (87). This analysis extracted candidate motions of the cytoplasmic portions of helices TM5 and TM6 and their linking loop from PDB: 3PXO (58), movements associated with TM7 (to which the retinal is covalently bound via Lys296) from PDB: 3CAP (60) and PDB: 1GZM as a reference resting structure of rhodopsin (59). We used these same PDB files and structural perturbations in the following analysis.

Structural fitting was performed by first generating a set of conformations using molecular dynamics simulations in GROMACS (55,56). During any set of simulations, we applied energetic restraints on the C*α* atoms of rhodopsin to restrain them about their resting conformation coordinates, C*α*_*Rho*_, or to drive them toward new coordinates according to the formula:

(3) $${C\alpha }_{Rho}\to {C\alpha }_{Rho}+{{\gamma \times \Delta C\alpha }_{TM5,6}+\delta \times \Delta C\alpha }_{TM7}\text{,}$$

where ΔC*α*_*TM*5,6_ is the candidate movement of the cytoplasmic side of TM5 and TM6, ΔC*α*_*TM*7_ is the candidate movement associated with TM7, and the variables *γ* and *δ* are varied stepwise to create a grid of 36 candidate movements, with nine values of *γ* associated with different amplitude movements of TM5 and TM6, and four values of *δ* associated with different amplitude movements of TM7. This procedure allows the protein to sample conformations about target structural perturbations, but does not necessarily mimic the natural flexibility of the protein. For each of these candidate structures, CRYSOL (61) was used to calculate the x-ray scattering intensities, *S*(*q*), from which the changes in x-ray scattering, Δ*S*(*q*), were calculated by subtracting the equivalent calculations from the restrained resting rhodopsin trajectory. Additional details of this procedure are described in the materials and methods.

**Figure 6:**
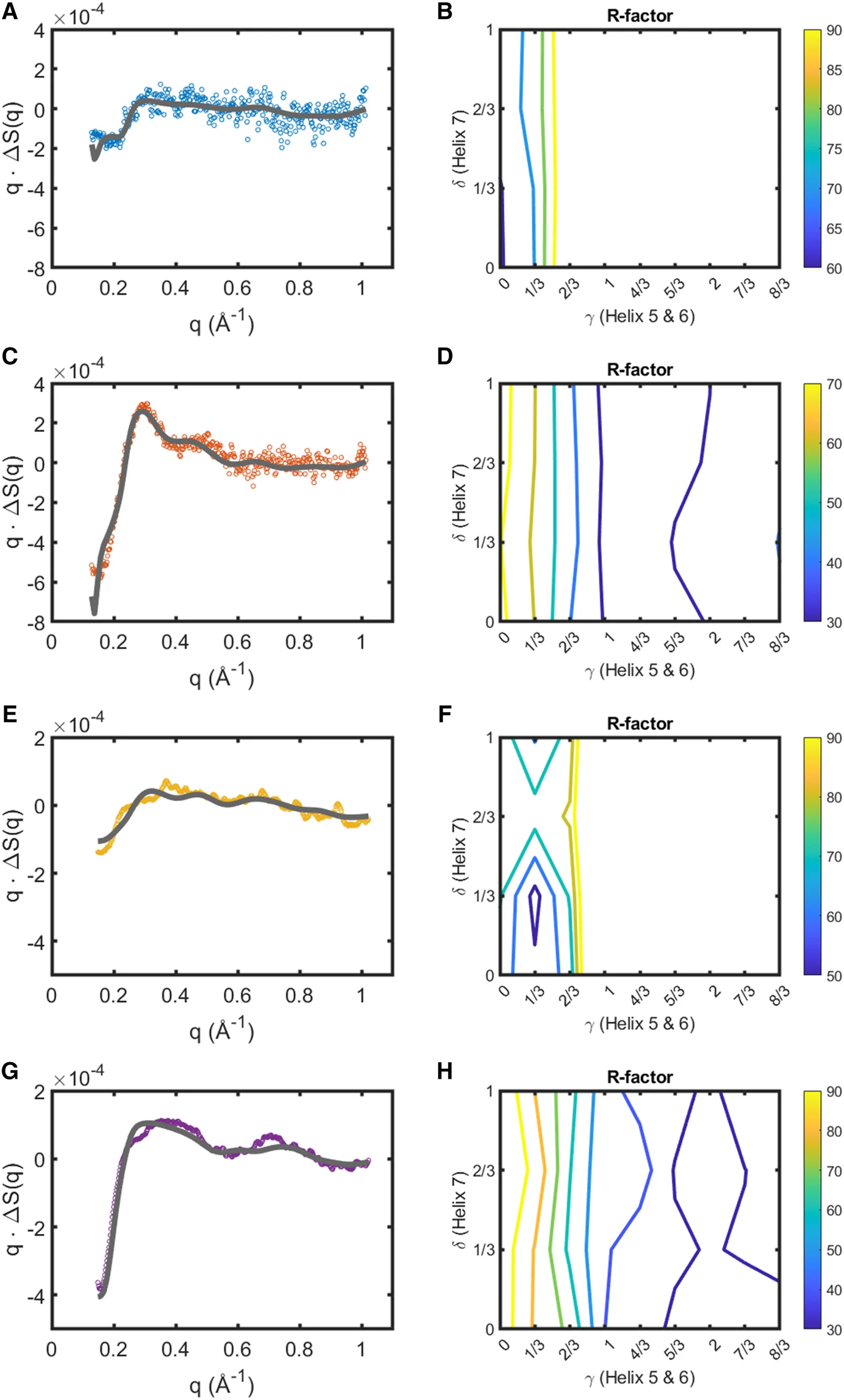
Structural modeling of TR-XSS data to recover structural changes in visual rhodopsin solubilized in DDM or CHAPS. (*A*) State 1 basis spectrum (BS1_DDM_) extracted from TR-XSS data (*blue*) recorded from visual rhodopsin in DDM with the best-fit prediction recovered from structural modeling superimposed (*gray line*). (*B*) Contour plot showing the *R*-factor recovered as the parameters *γ* and *δ* are varied during structural fitting as described in (13). (*C*) State 2 basis spectrum (BS2_DDM_) extracted from TR-XSS data (*red*) recorded from visual rhodopsin in DDM with the best-fit prediction recovered from structural modeling superimposed (*gray line*). (*D*) Contour plot showing the *R*-factor recovered as the parameters *γ* and *δ* are varied during structural fitting. (*E*) State 1 basis spectrum (BS1_CHAPS_) extracted from TR-XSS data (*mustard*) recorded from visual rhodopsin in CHAPS with the best-fit prediction recovered from structural modeling superimposed (*gray line*). (*F*) Contour plot showing the *R*-factor recovered as the parameters *γ* and *δ* are varied during structural fitting. (*G*) State 2 basis spectrum (BS2_CHAPS_) extracted from TR-XSS data (*purple*) recorded from visual rhodopsin in CHAPS with the best-fit prediction recovered from structural modeling superimposed (*gray line*). (*H*) Contour plot showing the *R*-factor recovered as the parameters *γ* and *δ* are varied during structural fitting.

Results from this procedure are illustrated in Fig. 6. Optimal fits to the difference x-ray scattering data are shown in gray, whereas the experimental data are shown as circles and are colored according to Fig. 4. Most critically, structural fitting is able to capture all of the features associated with BS2_DDM_ from DDM and BS2_CHAPS_ from CHAPS. This establishes that, despite the very strong influence of the surrounding detergent micelle (Fig. 1), the theoretical formalism (13) is sufficiently robust to encompass these changes. The quality of these fitting procedures is quantified by a weighted *R*-factor (Eq. 1, materials and methods) which yields a discrepancy between the difference XSS data and model of 22.7% for BS2_DDM_ (Fig. 6 *C*) and 24.6% for BS2_CHAPS_ (Fig. 6 *G*). These values provide a good fit against difference x-ray scattering data given the limitations of the formalism and the fact that samples of visual rhodopsin could not be cycled, limiting the signal/noise ratios in these data. In this formalism, the point when the detergent micelle cross term falls to zero is defined by the midpoint, *q*_*pm*_, and width, Δ*q*_*pm*_, of the error function, erf(*q*_*pm*_,Δ*q*_*pm*_), of Eq. 2. For BS2_DDM_ the optimal parameters were *q*_*pm*_ = 0.28 Å^−1^ and Δ*q*_*pm*_ = 0.29 Å^−1^, whereas the corresponding values for BS2_CHAPS_ were *q*_*pm*_ = 0.23 Å^−1^ and Δ*q*_*pm*_ = 0.17 Å^−1^. These domains have also been identified as important in small-angle x-ray scattering studies of membrane proteins in detergent micelles (64,65). In both cases, the quality of the fit to BS1 is not as good as for BS2, since the signal/noise ratio is much weaker due to the smaller amplitude of the extracted difference x-ray scattering features. As such, the weighted *R*-factor obtained for BS1_DDM_ was 58.8% (Fig. 6 *A*), and the weighted *R*-factor for BS1_CHAPS_ was 46.0%. (Fig. 6 *E*). These relatively high *R*-factors reflect the very low signal associated with these first basis spectra and consequently the signal/noise ratio is not favorable.

Contour plots showing the surface of weighted *R*-factor values about the best fits indicate the confidence of the fitting procedure in finding a local minimum, and the relative dependence of the overall fit on the parameters *γ* and *δ*. From Fig. 6, *B*, *D*, *F*, and *H* it is apparent that this analysis is sensitive to the motions of TM5 and TM6, but much less sensitive to perturbations of TM7. This is reflected by the presence of a clear valley in the plotted contour as the parameter *γ* varies, whereas this surface is relatively insensitive to changes in the value of *δ*. This finding is consistent with an earlier analysis of TR-XSS data from visual rhodopsin in native membranes, where it was observed that the fitting procedure was sensitive only to perturbations in TM5 and TM6 (17). Moreover, taking *γ* = 1 to correspond to the structural perturbation associated with differences between PDB: 3PXO and 1GZM, it is apparent from this contour surface representation that the structural perturbations associated with BS2 for both DDM and CHAPS are larger than those reported from earlier crystallographic studies.

**Figure 7:**
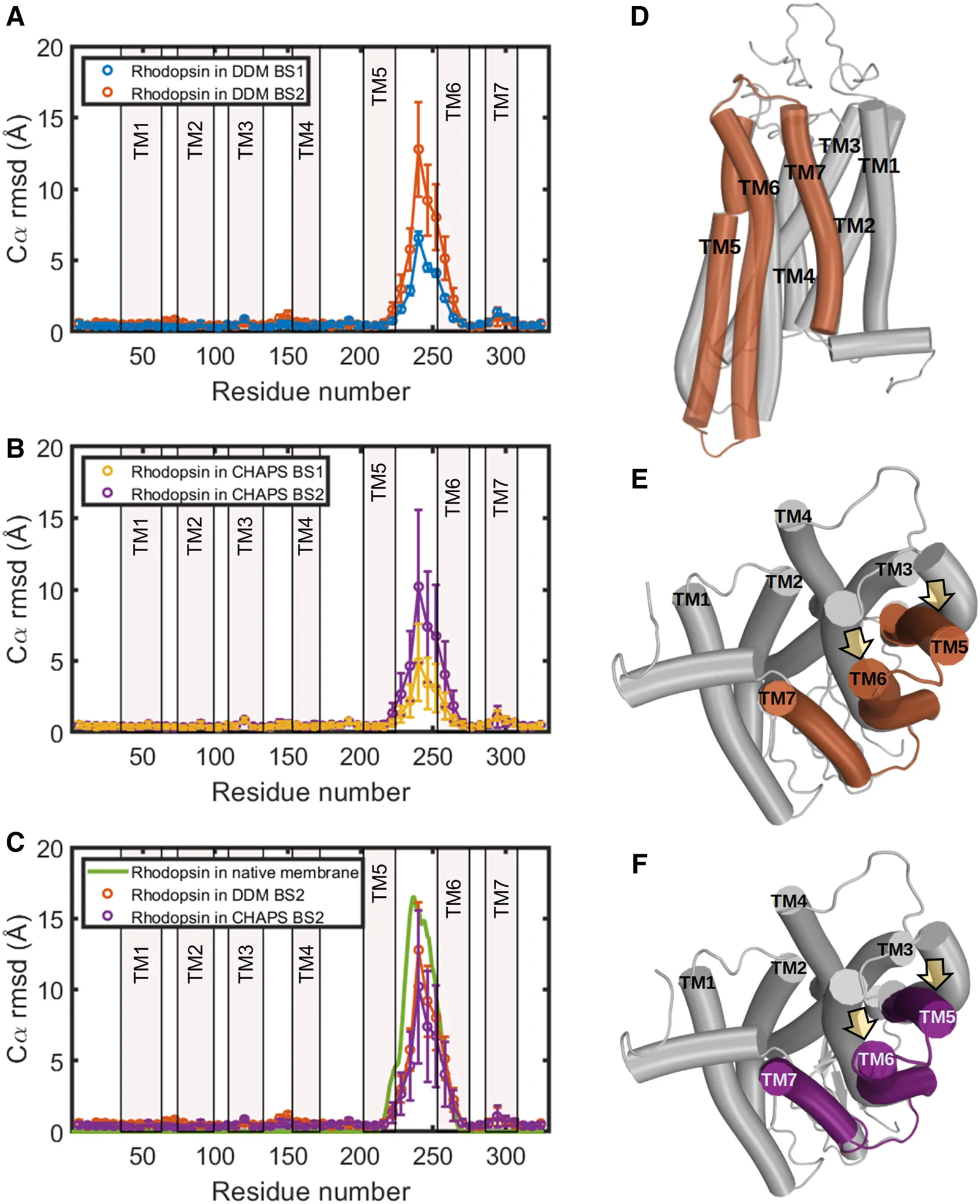
Light-induced structural changes in visual rhodopsin. (*A*) Root mean-square displacements (rmsd) of Cα-atoms of rhodopsin and corresponding uncertainty bars resulting from an ensemble of conformations that that best fit the difference x-ray scattering data. Blue: the mean displacements of Cα-atoms when fitting BS1_DDM_ (Fig. 6*A*). Red: the mean displacements of Cα-atoms when fitting BS2_DDM_ (Fig. 6*C*). (*B*) rmsd of Cα-atoms and uncertainty bars resulting from the best fit of the difference x-ray scattering data to BS1_CHAPS_ (*mustard*, Fig. 6*E*) and BS2_CHAPS_ (*purple*, Fig. 6*G*). (*C*) Superposition of the rmsd of Cα-atoms of rhodopsin and uncertainty bars resulting from the best fit the difference x-ray scattering data to BS2_DDM_ (*red*), BS2_CHAPS_ (*purple*), and the best-fit model against native membranes (17) (*green*, no error bars). (*D*) Superposition of the structure of visual rhodopsin in its resting conformation (*light gray*) and after fitting to BS2_DDM_ (*red*) when viewed from the plane of the membrane. (*E*) Superposition of the structure of visual rhodopsin in its resting conformation (*light gray*) and after fitting to BS2_DDM_ (*red*) when viewed from the cytoplasm perpendicular to the membrane. (*F*) Superposition of the structure of visual rhodopsin in its resting conformation (*light gray*) and after fitting to BS2_CHAPS_ (*purple*) when viewed from the cytoplasm perpendicular to the membrane.

Structural fitting results are better represented by plotting the root mean-square displacements (rmsd) of C*α* coordinates for the activated conformation relative to the resting conformation, and associated uncertainty bars extracted from an ensemble of structures that are close to the best-fit model (materials and methods). Structural fitting against BS1_DDM_ suggests that TM5 and TM6 undergo some rearrangements already on the microsecond timescale, with considerably extended motions for BS2_DDM_ for longer time delays (Fig. 7 *A*). Similar secondary structural rearrangements are recovered for structural fitting against BS1_CHAPS_ and BS2_CHAPS_ (Fig. 7 *B*) despite the very different basis spectra (Fig. 6). Since we recovered good signal/noise ratios in the difference x-ray scattering data for BS2_DDM_ and BS2_CHAPS_, it is quite striking that, despite very large differences in the x-ray scattering spectra, the modeled conformational changes agree within experimental errors (Fig. 7 *C*). These rearrangements are somewhat larger than those extracted as candidate movements from the crystallographic structures, while still agreeing within the estimated error bars. When comparing with structural results recovered when fitting against TR-XSS data recorded from visual rhodopsin in native disk membranes (17), the structural models recovered here show modestly smaller movements than previously modeled, but again agree within the estimated coordinate errors. In this context, it is important to appreciate that the earlier fitting against difference x-ray scattering data used rigid body alignments and imposed a B-factor term to damp oscillations in the difference x-ray scattering data at high angle (17), rather than sampling from multiple restrained molecular dynamics trajectories. It therefore seems plausible that the additional step of generating an ensemble of structures using restrained molecular dynamics simulations in GROMACS reduces the amplitude of the structural perturbation, since the simulations will also be restrained to achieve physically reasonable protein conformations (Fig. 7, *D*–*F*). When comparing the optimal fits to the x-ray scattering data against the candidate motions from x-ray crystallography, we find that light-induced α-helical motions in solution are approximately 35% larger than those observed in the corresponding crystallographic structures. The amplitude of α-helix perturbations observed in the crystal structure of the β_2_-adrenergic receptor in complex with its G-protein (91) were also larger than the amplitude of similar motions reported in earlier studies of rhodopsin (60,86).

## Discussion

In this study we aimed to develop the theoretical formalism for incorporating the influence of detergent micelles on difference x-ray scattering data by building out from an earlier TR-XSS study of conformational changes in visual rhodopsin in native disk membranes (17). Our experimental data show very different x-ray scattering basis spectra for the conformational states of visual rhodopsin when solubilized in DDM and CHAPS (Fig. 5), yet structural fitting against the experimental data could successfully capture all the major features of these experimental data (Fig. 6). Moreover, the major conclusion of an earlier TR-XSS study of visual rhodopsin in native disk membranes (17) was reproduced, since structural fitting against difference x-ray scattering data again suggest that the conformational state of fully activated rhodopsin undergoes larger α-helical rearrangements than were observed in the crystallographic structures of activated rhodopsin (58,60,81,82,83,84,85,86,87,88). However, in this study the modeled movements of TM6 and TM7 are not as extensive as previously modeled against TR-XSS data (17) (Fig. 7 *C*). It seems that the extent of α-helical motions may be somewhat restrained by energy considerations when using the molecular dynamics package GROMACS to create an ensemble of candidate structures, rather than when fitting against rigid body α-helical motions, as were utilized in the previous study. As previously argued, the larger movements associated with the metarhodopsin II state relative to earlier crystal structures of the apoprotein opsin (60,86,89) may be due to the visual rhodopsin becoming more compact once retinal is hydrolyzed and lost (Fig. 1 *A*), and therefore opsin may indeed have smaller structural perturbations than metarhodopsin II.

With machine learning approaches to structural prediction proving to be extremely powerful (1,2,39), experimental structural biology is at a crossroads. The scientific community’s confidence in the accuracy of structural predictions will grow as the field becomes increasingly familiar with applying these tools to biological questions. Machine learning tools also provide an ensemble of predicted protein structures and this information provides a rich source for extracting hypotheses regarding conformational changes that arise along enzymatic or signaling trajectories. These developments will facilitate modeling macromolecular conformational changes against TR-XSS data, since the weakest point of TR-XSS studies is structural modeling. Integral membrane proteins perform a diverse set of functional tasks that are essential to the cell. Knowledge of their reaction pathways is essential to understand how membrane protein enacted functions are performed in living organisms. In this work we have validated experimentally the theoretical prediction that difference x-ray scattering curves from integral membrane proteins will be strongly influenced by the solvent contrast of the surrounding detergent micelle (13). This validation should allow future studies of conformational changes in other membrane proteins to be examined, improving confidence in the study of a diverse selection of integral membrane protein targets and helping to expand the systems of interest to incorporate more proteins that are not naturally light sensitive (32,33,34).

## Data and code availability

• The data and in-house code that support the findings of this study are publicly available as of the date of publication in GitHub at https://github.com/Neutze-lab/Rhodopsin_Detergent.
• Any additional information required to reanalyze the data reported in this paper is available from the corresponding author upon request.

## Acknowledgments

R.N. acknowledges funding from the 10.13039/501100004359Swedish Research Council (grant no. 2015-00560) and the European Union’s 10.13039/501100007601Horizon 2020 research and innovation program (grant agreement no. 789030). M.F.B. acknowledges financial support from US 10.13039/100000001National Science Foundation grants CHE 1904125, DMR 2350337, and MCB 1817862. G.F.X.S. acknowledges the 10.13039/501100001711Swiss National Science Foundation through project grant SNF_310030B_173335. We thank Suchithranga M.D.C. Perera and Andrey V. Struts for assistance with the rhodopsin preparations and sample characterizations in CHAPS detergent.

## Author contributions

R.N., G.F.X.S., M.F.B., and V.P. conceptualized this work. X.X., T.G., and N.V. prepared samples under the guidance of M.F.B., V.P., and G.F.X.S. M.N.P., M.S., M.W., and M.L. prepared the beamline ID09 of the ESRF for these experiments and supported data collection. D.S., X.X., T.G., N.V., R.B., O.B., V.P., and R.N. participated in data collection at the ESRF. D.S. processed and decomposed the x-ray scattering data with support from R.N. D.S. and L.O. performed guided molecular dynamics simulations. Structural refinement was performed by R.N. with support from L.O. and D.S. R.N. wrote the article with support from L.O. and input from D.S., V.P., M.F.B., G.F.X.S., and M.L. R.N., M.F.B., and G.F.X.S. secured funding for this work.

## Declaration of interests

The authors declare no competing interests.
